# Oxidative Stress Triggers a Pivotal Peptide Linked to Alzheimer’s Disease

**DOI:** 10.3390/ijms252212413

**Published:** 2024-11-19

**Authors:** Nikki Evans, Kashif Mahfooz, Sara Garcia-Rates, Susan Greenfield

**Affiliations:** Neuro-Bio Ltd., Building F5, Culham Campus, Abingdon OX14 3DB, UK; nikki.evans@neuro-bio.com (N.E.); sara.garciarates@neuro-bio.com (S.G.-R.); susan.greenfield@neuro-bio.com (S.G.)

**Keywords:** oxidative stress, Alzheimer’s disease, AChE, T14, NBP14

## Abstract

An aberrant recapitulation of a developmental mechanism driven by a 14 mer peptide (‘T14’) derived from acetylcholinesterase (AChE) has been implicated in Alzheimer’s disease. T14 was suggested as an upstream driver of neurodegeneration due to its ability to stimulate the production of phosphorylated tau and amyloid beta. The activation of this mechanism in adulthood is thought to be brought upon by insult to the primarily vulnerable subcortical nuclei. Here, we show that oxidative stress, induced by high glucose and confirmed by an analysis of antioxidant enzyme mRNA expression, increased the levels of T14 peptide in PC12 cells. This increase in T14 corresponded with an increase in the mRNA expression of AChE and a decrease in the cell viability. The increase in T14 could be blocked by the cyclic form of T14, NBP14, which prevented any cytotoxic effects. These observations suggest that oxidative stress can directly trigger the inappropriate activation of T14 in the adult brain through the upregulation of *Ache* mRNA.

## 1. Introduction

Alzheimer’s disease (AD) is a neurodegenerative disease that is the leading cause of dementia worldwide [1]. Oxidative stress, characterised by an imbalance between reactive oxygen species (ROS) and the cellular antioxidant defence system, is a phenomenon that increases in the ageing brain [2] and has been implicated in AD [2,3,4]. Reduced or compromised levels of antioxidant enzymes and elevated ROS generated from mitochondrial dysfunction in AD enhance oxidative stress, which causes damage to DNA, lipids, and proteins [3,4]. This is thought to exacerbate the formation of amyloid beta and neurofibrillary tangles, which are hallmark features of AD, since it occurs prior to amyloid beta plaque formation and tau phosphorylation [5], and the prevention of oxidative stress with antioxidant supplementation precludes the amyloid-beta-induced phosphorylation of tau [6]. Moreover, increased mitochondrial DNA oxidation, which is an indicator of mitochondria oxidative stress, is one of the first markers of AD [3]. To mimic these conditions in vitro, high glucose was used in this study, as it was shown to activate the mitochondrial electron transport chain [7] and increase oxidative stress [8,9,10].

The 14 mer peptide ‘T14’, with similarity to amyloid beta, is derived from acetylcholinesterase (AChE) through proteolytic cleavage [11] and has the amino acid sequence AEFHRWSSYMVHWK [12]. It shows independent bioactivity in a range of in vitro and ex vivo preparations [12,13,14], and acts as a signalling molecule via the α7 nicotinic acetylcholine receptor to increase the calcium influx [15]. It has a trophic role during development [12]; however, when aberrantly activated during adulthood, it has cytotoxic effects through calcium-induced excitotoxicity [16]. Additionally, T14 levels were found to be increased in AD brains [11].

It is well established that there is an altered expression of AChE in AD [17,18,19]. The combination of elevated oxidative stress, altered AChE expression, and upregulated T14 levels in AD prompted the hypothesis that there could be a link between these three factors, and that oxidative stress could be the trigger for the increased T14 levels through the upregulation of *Ache* mRNA expression. Hence, we first investigated high glucose as an inducer for oxidative stress in PC12 cells through measuring cell viability and antioxidant enzyme mRNA expression. We then looked at the impact of elevated oxidative stress on the mRNA expression of AChE. Using Western blot detection, we measured the T14 levels to determine whether it was upregulated by oxidative stress. Finally, we co-treated the cells with glucose and NBP14 to examine whether NBP14 could reverse the changes associated with high-glucose-induced oxidative stress.

## 2. Results

### 2.1. Effect of High Glucose on Cell Viability

**Figure 1:**
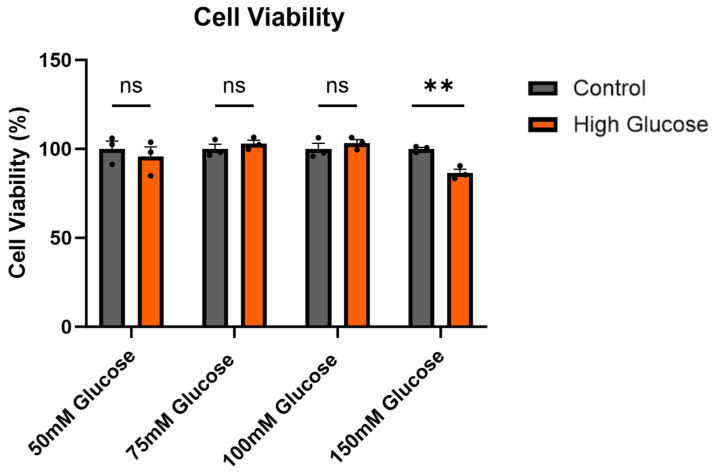
The cell viability was reduced with 150 mM glucose. The PC12 cells were treated with 50 mM glucose, 75 mM glucose, 100 mM glucose, and 150 mM glucose for 24 h before the cell viability was determined. The bars represent the mean number of live cells expressed as a percentage relative to the control. All bars are presented as the mean ± SEM, where n = 3. Student’s *t*-test. ** *p* < 0.01. ns: not significant.

The effects of glucose at 50 mM, 75 mM, 100 mM, and 150 mM for 24 h were measured in terms of cell viability. The results show a significant reduction in the cell viability of about 20% with 150 mM glucose compared with the control (Figure 1, unpaired *t*-test, *p* = 0.004203), which demonstrated cytotoxic effects at this concentration. Thus, 150 mM glucose was used for future experiments.

### 2.2. Induction of Oxidative Stress with High Glucose

**Figure 2:**
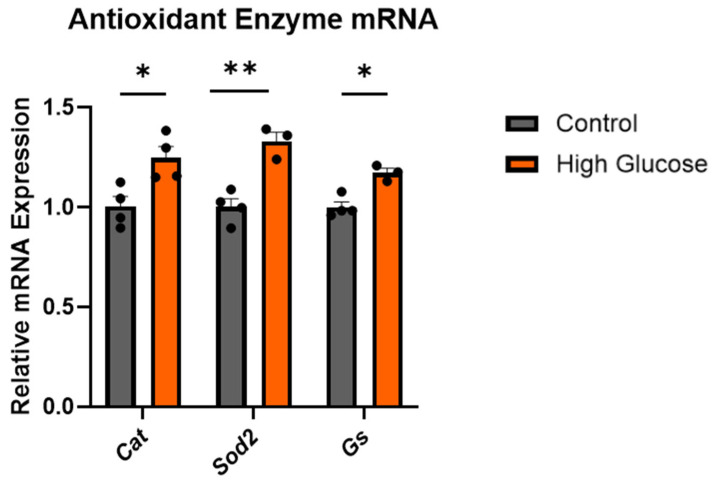
High glucose increased the expressions of *Cat*, *Sod2*, and *Gs*. Relative mRNA expression of the antioxidant enzymes: *Cat*, *Sod2*, and *Gs* in PC12 with 150 mM glucose treatment, normalised to the *Gapdh* mRNA expression. All bars are presented as the mean ± SEM, where n = 3–4. Student’s *t*-test. * *p* < 0.05. ** *p* < 0.01. *Cat*, catalase; *Sod*, superoxide dismutase; *Gs*, glutathione synthase.

To confirm that the cytotoxic effects with high glucose involved the oxidative stress pathway, the expression of the antioxidant enzymes catalase (*Cat*), superoxide dismutase 2 (*Sod2*), and glutathione synthase (*Gs*) were measured. As expected, there were significant increases in the expressions of all the antioxidant enzymes measured (Figure 2; unpaired *t*-test; *p* = 0.0189, *p* = 0.00310, and *p* = 0.00522, respectively). This confirmed that the treatment with 150 mM glucose was able to induce the oxidative stress pathway in these cells, as evidenced by the increased antioxidant defence response observed.

### 2.3. Oxidative Stress Increased Ache mRNA and T14 Expressions

**Figure 3:**
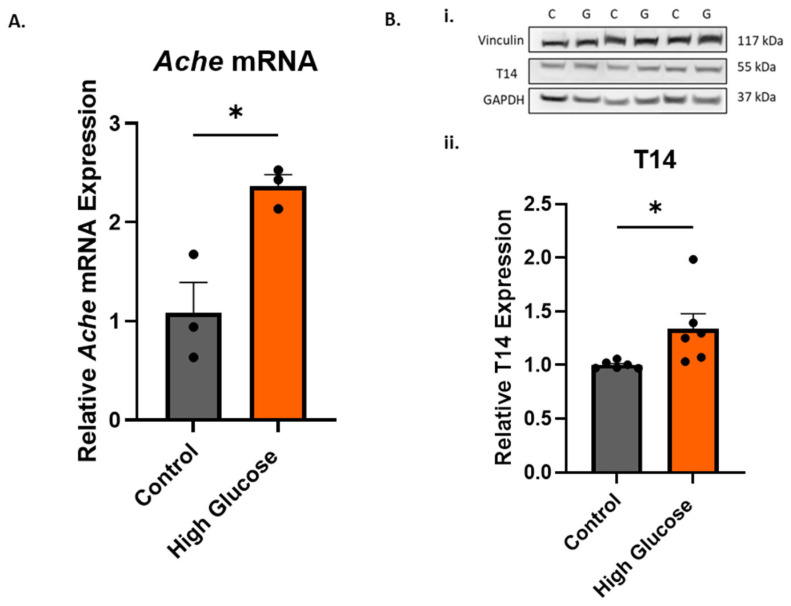
There was a significant increase in the *Ache* mRNA expression and T14 levels with high glucose. (**A**) Relative AChE mRNA expression, normalised to the *Gapdh* mRNA expression. (**Bi**) Representative Western blot of the high glucose (150 mM)-treated PC12 for vinculin (117 kDa), T14 (55 kDa), and GAPDH (37 kDa). C represents the control samples and G represents the high-glucose samples. (**Bii**) Relative T14 expression. The T14 expression was normalised to vinculin and GAPDH expression and expressed relative to the control. All bars represent the mean ± SEM, where n = 3–6. Student’s *t*-test. * *p* < 0.05.

To investigate the effects of the high-glucose-induced oxidative stress on *Ache* mRNA, qPCR was performed. A significant increase in the mRNA expression of AChE was found with high-glucose treatment (Figure 3A, unpaired *t*-test, *p* = 0.0179). To determine whether the increase in *Ache* mRNA expression was reflected in the T14 levels, a Western blot was then carried out. Interestingly, it was found that the increase in *Ache* mRNA was indeed accompanied by a significant increase in T14 levels with the glucose treatment (Figure 3(Bii), unpaired *t*-test, *p* = 0.0377).

### 2.4. NBP14 Reversed T14 Expression and Protected Against the Toxic Effects of High Glucose

**Figure 4:**
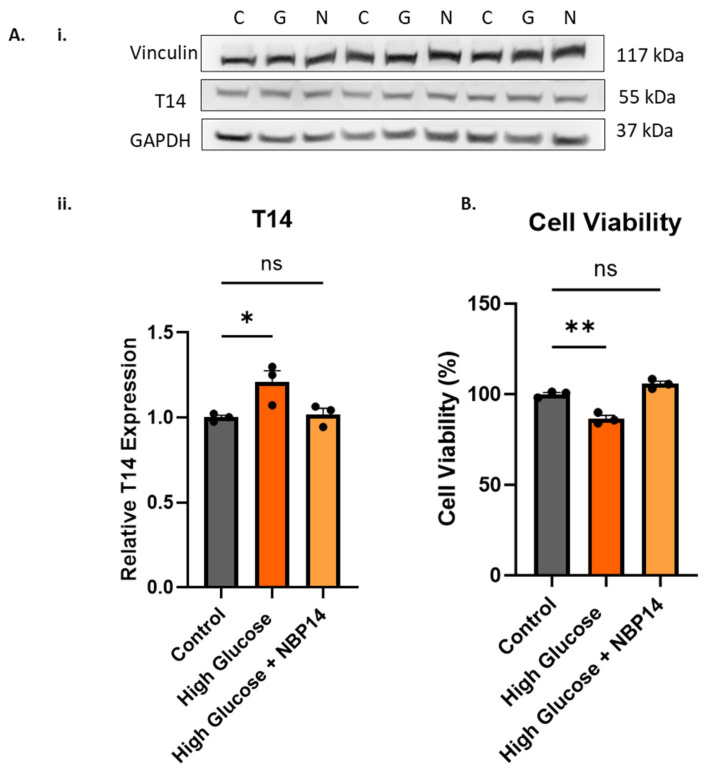
Co-treatment with NBP14 prevented the increase in T14 levels and reduction in cell viability seen with high glucose. (**Ai**) Representative Western blot of the high glucose (150 mM)-treated and high glucose (150 mM) + NBP14 (15 µM)-treated PC12 for vinculin (117 kDa), T14 (55 kDa), and GAPDH (37 kDa). C represents control samples, G represents high-glucose samples, and N represents high glucose + NBP14 samples. (**Aii**) Relative T14 expression normalised to vinculin and GAPDH expression and expressed relative to the control. (**B**) The cell viability of the PC12 cells treated with high glucose and high glucose + NBP14 for 24 h. The bars represent the mean number of live cells expressed as a percentage relative to the control. All bars represent the mean ± SEM, where n = 3. One-way ANOVA followed by Dunnett’s post hoc test. * *p* < 0.05. ** *p* < 0.01. ns: not significant.

To investigate whether NBP14 could prevent the increased production of T14 with high-glucose-induced oxidative stress, PC12 cells were treated with NBP14 in the presence of high glucose. Western blot detection for T14 expression showed changes across the groups (Figure 4(Aii), one-way ANOVA, F = 6.313, *p* = 0.0334, R^2^ = 0.6779), with a significant increase in the T14 levels with high glucose compared with the control (Dunnett’s post hoc test, *p* = 0.0326). When the cells were co-treated with NBP14 and high glucose, there was no change in the levels of T14 compared with the control (Dunnett’s post hoc test, *p* = 0.9502). Changes in the cell viability were also observed (Figure 4B, one-way ANOVA, F = 46.17, *p* = 0.0002, R^2^ = 0.9390). The cell viability was significantly reduced with high glucose (Dunnett’s post hoc test, *p* = 0.0011), while the co-treatment with NBP14 had no change in the cell viability compared with the control (Dunnett’s post hoc test, *p* = 0.0559).

### 2.5. Effects of NBP14 on Oxidative Stress

**Figure 5:**
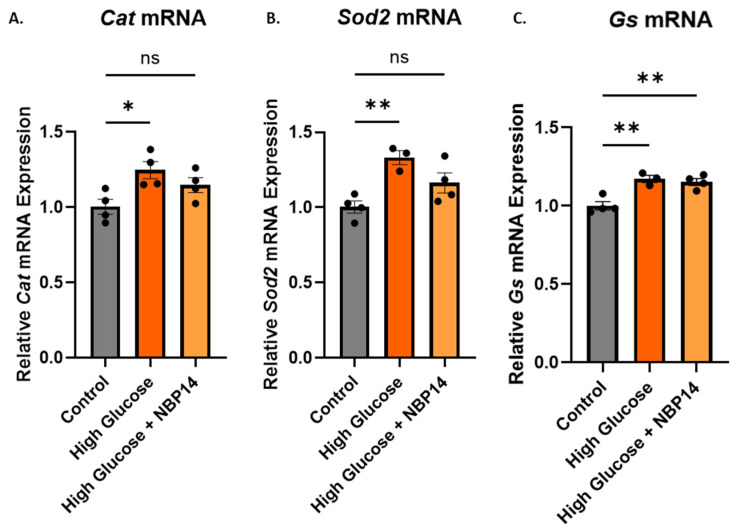
Co-treatment with NBP14 (15µM) attenuated the increase in *Sod2* and *Cat* mRNA expression with high glucose (150 mM) but had no effect on *Gs* mRNA expression. (**A**) Relative *Cat* mRNA expression. (**B**) Relative *Sod2* mRNA expression. (**C**) Relative *Gs* mRNA expression. All mRNA expressions were normalised to the *Gapdh* expression. All bars represent the mean ± SEM, where n = 3–4. One-way ANOVA followed by Dunnett’s post hoc test. * *p* < 0.05. ** *p* < 0.01. ns: not significant.

To determine whether the prevention of the T14 increase with NBP14 was due to oxidative stress, qPCR was performed for the antioxidant enzymes upregulated by high glucose: *Cat*, *Sod2*, and *Gs*. The detection for the three antioxidant enzymes showed changes across the groups (Figure 5A, one-way ANOVA, F = 5.417, *p* = 0.0286, R^2^ = 0.5462; Figure 5B, one-way ANOVA, F = 8.655, *p* = 0.01, R^2^ = 0.6839; Figure 5C, one-way ANOVA, F = 5.39, *p* = 0.0018, R^2^ = 0.7937) with a significant increase in the *Cat*, *Sod2*, and *Gs* mRNA expressions with 150 mM glucose (Dunnett’s post hoc test; *p* = 0.0175, *p* = 0.0059, and *p* = 0.0024, respectively). When the cells were co-treated with NBP14 and high glucose, there was a non-significant increase in the expression of *Cat* and *Sod2* (Dunnett’s post hoc test; *p* = 0.1466 and *p* = 0.1053, respectively), while for *Gs*, the co-treatment had a significant increase in its mRNA expression compared with the control (Dunnett’s post hoc test, *p* = 0.0031).

## 3. Discussion

### 3.1. Oxidative Stress and T14

A subcortical group of interconnecting nuclei, referred to as the ‘Isodendritic core’ [20] or ‘Global neurons’ [21], are the neurons that are the first to degenerate in AD [22]. These primarily vulnerable cells can be differentiated from other brain cells by their embryological provenance, i.e., the basal plate rather than alar plate, and most importantly, the ability to retain their developmental potential into maturity [21]. It was suggested that damage to the basal-plate-derived cells will trigger the inappropriate mobilisation of T14 in these cells [11], although the specific trigger has not been investigated yet. Undifferentiated PC12 cells retain their developmental potential by possessing the ability to differentiate into sympathetic-nerve-like cells through the application of neuronal growth factor (NGF) [23], mimicking these ‘global neurons’. Thus, we used undifferentiated PC12 cells in this study to allow for the close replication of these ‘global neurons’ in investigating whether oxidative stress could be one of the triggers for the aberrant mobilisation of the T14 mechanism.

To eliminate the effects of osmolarity from our glucose treatment, D-glucose was used, as its toxicity is independent of its osmolarity effect [8], isolating any changes seen from the direct effect of glucose on the cells. To confirm whether high-glucose-induced oxidative stress is similar to that in AD, the mRNA expression of some antioxidant enzymes altered in AD were measured. These were catalase (CAT), superoxide dismutase (SOD2), and glutathione synthase (GS) [24,25]. Reactive oxygen species are converted to hydrogen peroxide by SOD2 in the mitochondria, which is then broken down into hydrogen and water by CAT. GS is an enzyme responsible for the production of reduced glutathione (GSH), which, through a redox reaction with hydrogen peroxide catalysed by glutathione peroxidase, is able to detoxify hydrogen peroxide [26]. We observed an increase in the mRNA expression of the *Cat*, *Sod2*, and *Gs* with high glucose (Figure 2). However, in AD, the activities of these enzymes are generally reduced [24], although an increase in the mRNA expression was observed in the hippocampus and inferior parietal lobe [25]. This discrepancy in the results could be attributed to the type of sample (brain, plasma, blood) and the specific stage or region from which the sample was taken. Early oxidative stress may lead to an increase in the antioxidant enzyme expression, while extreme oxidative stress, particularly when the cellular defence system is compromised, might result in a decrease in the antioxidant enzyme expression. Nevertheless, the upregulation of antioxidant enzyme we observed with 150 mM glucose was a valid confirmation of oxidative stress, as it shows that the cellular defence system was mobilised.

**Figure 6:**
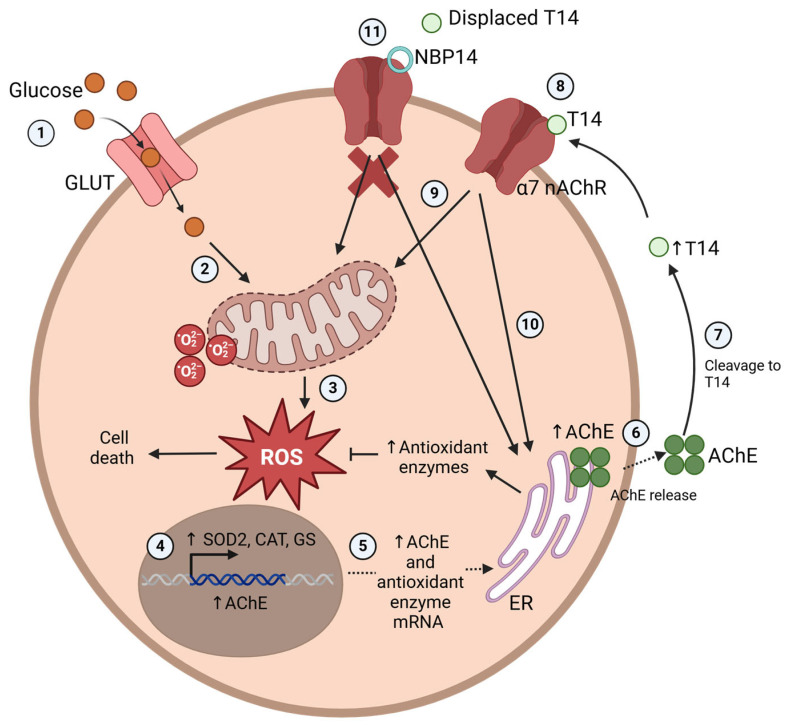
Proposed diagram of high-glucose-induced oxidative stress leading to elevated T14 levels and its downstream effects. (1) Glucose uptake occurs through GLUT transporters, which, for neuronal cells, are the GLUT1 and GLUT3 transporters [31]. (2) Once inside the cell, glucose activates increased mitochondrial electron transport chain activity [7], (3) generating ROS and contributing to the oxidative stress. (4) Elevated ROS activates the increased transcription of antioxidant enzymes and AChE. (5) The upregulated mRNA is transported outside the nucleus, where it is translated into protein at the endoplasmic reticulum. (6) Increased levels of produced AChE protein are released into the extracellular space, (7) where it is cleaved by proteases into T14 [32]. (8) T14 binds to the α7 nicotinic acetylcholine receptor [11,12], which (9) activates the mitochondria [14] downstream, further generating more ROS and contributing to cell death. (10) At the same time, AChE release from the reticulum is also stimulated [13], which is cleaved into T14, exacerbating the process. (11) NBP14 prevents T14 from binding to the α7 receptor by displacing it [30], thus blocking cascades activated by T14 and preventing the aberrant downstream effects by alleviating the generation of ROS from the mitochondria and AChE release. Figure created using BioRender.

The high-glucose-induced oxidative stress resulted in increased levels of *Ache* mRNA (Figure 3). Liu et al. (2016) [27] also observed increased ROS levels and AChE expression measured at the protein level when treating HT-22 cells with high glucose, confirming the phenomenon of high-glucose-induced oxidative stress affecting AChE. This observation could help explain why the increased *Ache* mRNA expression in AD [18,19] was associated with oxidative stress [19]. The T14 levels were also significantly increased with high glucose (Figure 3), which we suggest was from increased AChE release from the reticulum being cleaved into T14. Isik and Beydemir (2022) proposed that the increased AChE release from cells produced the toxic amyloid beta plaques, which led to neurodegeneration [19]. This increase in protein, rather than enzymatic activity, could perhaps be coming from AChE derived peptide T14, which was also proposed to enhance amyloid beta formation [14,16]. The decreased cell viability with high glucose (Figure 1) could be attributed to the direct effects of oxidative stress, i.e., ROS, but could also be complemented by the toxic effects of T14 [13]. T14 selectively activates mTORC1 [14,15], which affects mitochondrial dysfunction [28] and leads to more ROS, which elevates oxidative stress and cell death. T14 also causes a calcium influx [11,12], which is excitotoxic in high amounts. Furthermore, the feedforward mechanism of T14-induced compensatory AChE release [13] leads to more T14 production, exacerbating the whole process. The high-glucose-induced oxidative stress and its proposed downstream effects leading to higher T14 levels is summarised in Figure 6.

### 3.2. Protective Nature of NBP14

NBP14, a cyclic version of the T14 peptide [29], displaces T14 from the α7 nicotinic acetylcholine receptor [30], thus preventing the downstream effects activated by T14. We were able to confirm the protective effects of NBP14 in vitro, as when the cells were co-treated with NBP14, the increase in T14 levels and the decreased cell viability with high glucose were prevented (Figure 4). These results could be attributed to NBP14 displacing T14, and thus, preventing its downstream effects [13,14,30]. However, the question remained whether NBP14 could also prevent the toxic effects coming from oxidative stress. Thus, the expressions of the antioxidant enzymes were measured when the cells were co-treated with NBP14. The antioxidant enzyme expression was not reversed completely to baseline with the co-treatment of NBP14 (Figure 5); however, the expression was slightly less compared with the high-glucose group. There was a slight but not significant increase in *Sod2* and *Cat*, while a significant increase was found in *Gs* compared with the control. This effect could possibly be due to NBP14 blocking the ROS generation from the mitochondria through inhibiting the action of mTORC1 [14,28], but not preventing glucose from still activating the mitochondrial electron transport chain. Thus, the antioxidant enzymes were not reversed completely back to the baseline. Nevertheless, this observation suggests the possibility that NBP14 could possess antioxidant properties, thereby offering an exciting prospect to be further explored.

## 4. Materials and Methods

### 4.1. PC12 Cell Culture and Treatment

PC12 cells, derived from the pheochromocytoma of a rat adrenal medulla, commonly serve as a model for investigating AD pathology due to its neuronal properties [23]. Wild-type PC12 cells (catalog number: 88022401, Sigma, Merck, kGaA, Darmstadt, Germany) were cultured on 100 mM dishes (catalog number: CC7682-3394, Corning, Somerville, MA, USA, StarLab) coated with 2 mg/cm^2^ human placenta type IV collagen (catalog number: C5533, Sigma Merck, kGaA, Darmstadt, Germany). The cells were cultured in high-glucose DMEM (catalog number: 11574456, Thermo Fisher Scientific, Swindon, UK) supplemented with 10% heat-inactivated horse serum, 5% foetal bovine serum, penicillin/streptomycin (0.1 mg/mL), and amphotericin B (2.5 μg/mL). The cells were maintained in an incubator at 37 °C with 5% CO_2_ and 80% humidity, and the cell media was changed every 2 days. The cells were passaged when they reached 100% confluency by using a cell scraper and passing them through a needle to remove clumped cells. The cells used for experiments varied between passages 15 and 25. For treatment with glucose, the cells were plated onto collagenated 6-well or 12-well plates with a confluency of around 40%. Two days after plating, the cell media were replaced, and the high-glucose wells were supplemented with appropriate amounts of glucose solution. The glucose solution was made by dissolving D-(+)-Glucose (catalog number: G8270-1100G, Sigma, Merck, kGaA, Darmstadt, Germany) in distilled water to obtain a 500 mM stock solution, which was then passed through a sterile filter. For co-treatment with NBP14, NBP14 (dissolved in water) was added to the wells to reach a final concentration of 15 µM. The cells were harvested 24 h after the treatment.

### 4.2. Cell Viability

The cell viability was measured using Trypan Blue Solution, 0.4% (catalog number: 15250061, Gibco, Paisley, Scotland, UK). During the cell harvesting, after the cells were scraped in 1 mL of PBS and collected into an Eppendorf, 10 µL of this cell suspension was added to 10 µL of Trypan Blue Solution. Then, 10 µL of this mixture was added to a cell counter and the 4 quadrants were counted and averaged for the total number of alive and dead cells. The percentage of alive cells was calculated using number of alive cells/(number of alive + dead cells) × 100. This percentage was taken as the cell viability reading.

### 4.3. Real-Time Quantitative Polymerase Chain Reaction (RT-qPCR)

The total RNA from PC12 cells was extracted using the RNeasy Plus Mini Kit (Qiagen, Manchester, UK) according to the manufacturer’s instructions. An additional DNase step was followed using the RNase-Free DNase kit (Qiagen, Manchester, UK), in line with the manufacturer’s protocol. A total of 200–400 ng of RNA was used for the cDNA synthesis using the Applied Biosystems High-Capacity cDNA Reverse Transcription Kit (catlog number: 4368814, Waltham, MA, USA). RT-qPCR was run on a Q thermal cycler (Quantabio, Beverly, MA, USA) using PerfeCTa SYBR Green FastMix (catalog number: 95074-01, Quantabio, Beverly, MA, USA), as stated in the manufacturer’s protocol with primers from Table 1. Primers for *Cat* and *Sod2* were taken from [33] and *Gs* from [34]. The relative mRNA expression was quantified using the 2^−∆∆Ct^ method, as outlined in [35], with *Gapdh* as the reference gene. The Cq values generated from the Q software (v1.0.4, Quantabio, Beverly, MA, USA) were used instead of the Ct values.

### 4.4. Protein Extraction

PC12 cells were harvested from the plates and centrifuged at 500 g for 5 min at 4 °C to obtain a cell pellet. The cell pellets were lysed using a lysis mixture that consisted of RIPA lysis and extraction buffers (Thermo Fisher Scientific, Swindon, UK), a protease inhibitor cocktail (cOmplete™ ULTRA Tablets, Mini, EDTA-free, EASYpack Protease Inhibitor Cocktail, Roche, Basel, Switzerland), and a phosphatase inhibitor one (PhosSTOP, Roche). Cell lysates were then centrifuged at 13,000 rpm for 10 min at 4 °C and the resulting supernatant was collected in a new Eppendorf tube. The protein concentration was quantified using the Pierce 660 nm Protein Assay and interpolated from the standard curve (Thermo Fisher Scientific, Swindon, UK).

### 4.5. Western Blot

A total of 15 µg of protein was mixed with the correspondent amount of 10× Invitrogen Bolt Sample Reducing Agent (catalog number: B0009, Invitrogen, Waltham, MA, USA) and 4× Bolt Sample Buffer (catalog number: B0007, Invitrogen, Waltham, MA, USA) needed to obtain a 1× concentration and then heated at 60 °C for 10 min. Subsequently, they were loaded onto 10- or 15-well 4–12% Bolt Bis-Tris Plus Mini Protein Gels (catalog number: NW04120BOX, Invitrogen, Waltham, MA, USA). Electrophoresis to separate the proteins was undertaken at 150 V for 35 min using 20× Bolt MES SDS Running Buffer (catalog number: B000202, Invitrogen, Waltham, MA, USA) before the membranes were transferred onto polyvinylidene fluoride (PVDF) membranes (0.2 μM pore size; catalog number: 1620117 BioRad, Hercules, CA, USA) using 20× Bolt Transfer Buffer (catalog number: BT0061, Invitrogen, Waltham, MA, USA) supplemented with methanol. The transfer was conducted at 100 V for 65 min. The membrane was then blocked at room temperature for 1 h in a blocking solution that consisted of 5% skimmed milk in tris-buffered saline plus 0.05% Tween 20 (TBS-T). The membranes were then incubated at 4 °C with gentle agitation overnight in primary antibody added to the blocking solution. The primary antibody used against T14 (1:1000) was custom made by Genosphere Biotechnologies (Paris, France), while those for GAPDH (1:10,000; catalog number: ab181602) and vinculin (1:10,000; catalog number: ab129002) were purchased from Abcam (Cambridge, UK). After incubation, the membranes were washed three times with TBS-T for 5 min, before incubation with a secondary goat-anti-rabbit IgG H + L Horseradish peroxidase (HRP)-conjugated antibody (1:10,000; catalog number: G21234, Thermo Fisher Scientific, Swindon, UK) in a blocking solution. Incubation with a secondary antibody was carried out for 1 h at room temperature with gentle agitation. The membranes were then washed with TBS-T three times for 5 min, followed by a final wash with tris-buffered saline (TBS) for 5 min. Visualisation of the proteins was performed using an enhanced chemiluminescence-based detection kit (catalog number: 1705061, BioRad, Hercules, CA, USA) according to the manufacturer’s protocol and a CCD Camera (G-Box, Syngene, Cambridge, UK) gel system. Protein expression was measured based on the band intensities, which were quantified using the ImageJ software version 1.54. The T14 expression was normalised to that of the geometric mean of GAPDH and vinculin.

### 4.6. Statistical Analysis

All data were expressed relative to the control and the error represented as the standard error of the mean (SEM). A Shapiro–Wilk test was used to check for the normality of the data, which resulted in parametric tests being conducted throughout. If there were two groups, an unpaired *t*-test was performed. If there were more than two groups, a one-way ANOVA followed by Dunnett’s post hoc test was conducted. All analyses were performed in GraphPad PRISM version 10.3.0, with *p* < 0.05 being statistically significant throughout.

## 5. Conclusions

This study demonstrated that oxidative stress upregulated the T14 peptide. Thus, when oxidative stress is abnormally elevated in the adult brain, as in the case of AD, it may act as a stimulus for the aberrant reactivation of T14 by upregulating the *Ache* mRNA expression. Therefore, NBP14 could have the potential to serve as a therapeutic agent for blocking the cytotoxic effects of oxidative stress through the T14 blockade and potential antioxidant.

## 6. Patents

The NBP14 used in this study was covered by an international patent application: “WO2015/004430” (Family 1-NBP-14).

## Figures and Tables

**Table 1:** The primers used in the RT-qPCR.

Gene	Forward Primer Sequence (5′-3′)	Reverse Primer Sequence	Product Size (bp)
*Ache*	GGTTCTCCTTCGTGCCTGT	AGCCCTCATCCTTCACCAC	113
*Cat*	GGCAGCTATGTGAGAGCC	CTGACGTCCACCCTGACT	373
*Gapdh*	GGGCTCTCTGCTCCTCCCTGT	CAGGCGTCCGATACGGCCAAA	119
*Gs*	CACCAGCTGGGGAAGCATCT	GGTGAGGGGAAGAGCGTGAA	162
*Sod2*	CTGAGGAGAGCAGCGGTCGT	CTTGGCCAGCGCCTCGTGGT	371

## Data Availability

The data supporting this study are available within this paper.
